# Proposal for Describing Procedures to Correct Varicocele. A New Terminology

**DOI:** 10.3389/fped.2014.00047

**Published:** 2014-05-30

**Authors:** Ricardo González

**Affiliations:** ^1^Pediatric Surgery and Urology, Auf der Bult Kinder und Jugendkrankenhaus, Hannover, Germany; ^2^Urology and Pediatric Surgery, Universitätsmedizin Berlin – Charité Virchow Klinikum, Berlin, Germany

**Keywords:** varicocele, varicocele operations, Ivanissevich procedure, Palomo procedure, eponyms

Although the use of eponyms to describe specific surgical operations is often discouraged (1), the custom is so deeply ingrained in medical writing that readers inevitably encounter them. Therefore, for the sake of clarity in communications, one should strive to be correct in their use.

A classic case of perpetuation of misinformation is the use of eponyms to describe the operations performed to correct varicocele.

Most seemingly authoritative reviews repeatedly state that the eponym “Ivanissevich procedure” refers to the transinguinal ligation of the spermatic vein. This is a mistake and is repeated through the published literature since access to the original papers may require an additional effort rarely made in this era of computerized searches (2–5).

## Historical Review

**Figure 1:**
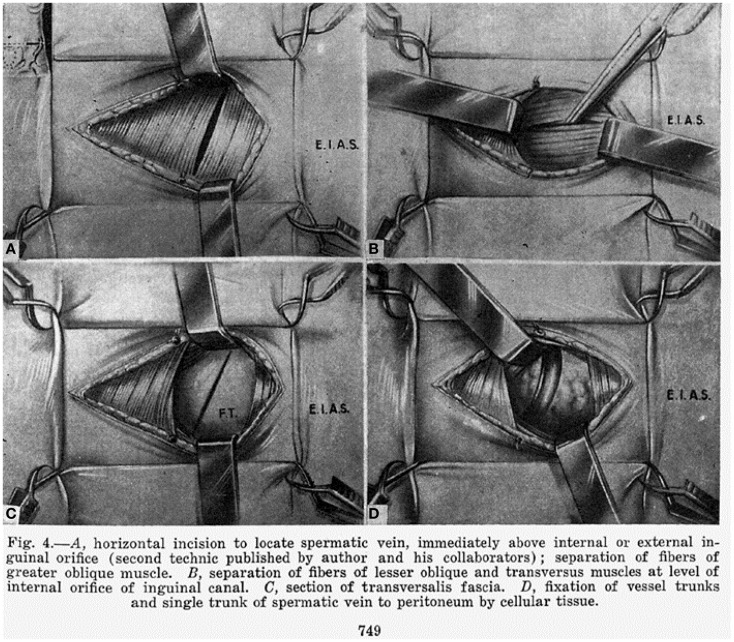
**The figure from Ivanissevich’s 1960 paper (7) clearly shows that the incision (first described in the 1918 paper) is medial to the anterior superior iliac spine, thus a suprainguinal or retroperitoneal approach (presented at the 24th Annual Congress of the European Society for Paediatric Urology, Genoa, Italy)**. With permission from International College of Surgeons.

In 1918, Dr. Oscar Ivanissevich working in Buenos Aires described the anatomy of the spermatic vein and proposed a suprainguinal approach to spermatic vein ligation (6). The rationale for this approach was to ligate the vein where it was most likely to have a single trunk. In 1960, he reported his experience with more than 4000 cases using the suprainguinal approach in an English language journal and provided detailed illustration of his technique (7) (Figure 1).

It was actually Bernardi, a disciple of Ivanissevich who advocated a transinguinal approach to spermatic vein ligation (8). In his 1960 article, Ivanissevich is critical of Bernardi’s transinguinal approach but admits that for inexperienced surgeons it might be easier than the retroperitoneal. He further stated that one should still strive to ligate the vein above the internal inguinal ring.

In 1949, Palomo described the ligation of the spermatic vein and artery through a retroperitoneal approach. He reported preliminary results but a more definitive article never followed. Although he gives no credit to Ivanissevich for the approach, being Spanish-speaking, it is unlikely that he was not aware of Ivanissevich’s work. Some authors have mistakenly applied the term “modified Palomo” procedure for the retroperitoneal approach preserving the artery (9) when in reality this is no other than the procedure described by Ivanissevich.

A possible reason for these errors is that the original articles are no easy to locate and there are no abstracts attached to the titles in PubMed and authors quote others who also have not personally read the references.

In summary, the correct terminology, based on a review of the full-text original articles is:

Suprainguinal approach ligation of only the vein: Ivanissevich.
Suprainguinal approach ligation of artery, vein, and lymphatics: Palomo.
Inguinal approach with ligature of the vein or veins: Bernardi.

However, considering that there are approaches other than open operations that are gaining popularity such as the transvascular venous occlusion (10) and laparoscopic vein ligation (11), and for the sake of clarity, I propose the following terminology to describe varicocele operations which includes the description of the approach, what is done, and the use of optical aids.

**Approach:**

Subinguinal
Inguinal
Suprainguinal (retroperitoneal)
Laparoscopic
Transvenous

**What is occluded:**

Vein
Artery and vein
Artery, vein, and lymphatics

**Optical magnification:**

None
Loupes
Microscope
Laparoscopy

If this terminology is accepted and widely adopted, publications addressing this still controversial topic would be easier to interpret.

## Conflict of Interest Statement

The author declares that the research was conducted in the absence of any commercial or financial relationships that could be construed as a potential conflict of interest.
